# DTI-ALPS index and cognitive impairment in cerebral small vessel disease: a systematic review and meta-analysis

**DOI:** 10.3389/fnagi.2026.1853704

**Published:** 2026-08-26

**Authors:** Xiao-xue Zeng, Ming-xi Xu, Xia He, Xia-lian Huang, Ying-ying Yang, Feng-le Mao, Fu-li Qin, Yan-qiu Wang

**Affiliations:** 1 Chengdu University of Traditional Chinese Medicine, Chengdu, China; 2 Sichuan Provincial Rehabilitation Hospital, Chengdu, China

**Keywords:** cerebral small vessel disease, cognitive impairment, DTI-ALPS index, glymphatic system, meta-analysis

## Abstract

**Objective:**

The diffusion tensor image analysis along the perivascular space (DTI-ALPS) index is a non-invasive diffusion tensor imaging-based metric proposed to reflect glymphatic processes. This meta-analysis aimed to evaluate the association of the DTI-ALPS index with cerebral small vessel disease (CSVD) and CSVD-associated cognitive impairment.

**Methods:**

This meta-analysis was conducted in accordance with the Preferred Reporting Items for Systematic Reviews and Meta-Analyses (PRISMA) guidelines. Eight databases were systematically searched from inception up to February 1, 2026. Study selection and data extraction were performed independently by two reviewers. Study quality was assessed using the Newcastle–Ottawa Scale adapted for cross-sectional studies (NOS-xs). Pooled effect sizes were expressed as weighted mean differences (WMDs) with 95% confidence intervals (CIs). Heterogeneity was evaluated using Cochran’s Q (χ^2^ test) and the I^2^ statistic, while publication bias was assessed by funnel plots and Egger’s regression test.

**Results:**

A total of 14 studies involving 2,149 participants were included. The median NOS-xs score was 8 (range, 6–9). Heterogeneity was observed across comparisons, with I^2^ values of up to 80%. Publication bias was assessed only for the comparison between CSVD and healthy controls, and no evidence of publication bias was detected (Egger’s test, *P* = 0.189). The pooled analysis showed that the DTI-ALPS index was significantly lower in patients with CSVD than in healthy controls (WMD = -0.12, 95% CI: -0.15 to -0.10). Furthermore, compared with cognitively normal participants, the DTI-ALPS index was significantly reduced in patients with CSVD-associated cognitive impairment (CSVD-CI), mild cognitive impairment (CSVD-MCI), and vascular dementia (CSVD-VaD) (all *P* < 0.05). However, no significant difference was observed between the CSVD-MCI and CSVD-VaD groups (WMD = -0.03, 95% CI: -0.08 to 0.02; *P* = 0.19).

**Conclusion:**

Patients with CSVD exhibit reduced DTI-ALPS indices, which are associated with the presence of cognitive impairment. Reduced DTI-ALPS indices were observed in patients with CSVD-MCI compared with cognitively normal patients with CSVD, indicating that this association is present at the MCI stage. However, because no significant difference was observed between the CSVD-MCI and CSVD-VaD groups, the value of the DTI-ALPS index for disease staging remains uncertain and requires confirmation in future longitudinal studies.

**Systematic Review Registration:**

https://www.crd.york.ac.uk/prospero/ #recordDetails, identifier [CRD420261322688].

## 1 Introduction

Cerebral small vessel disease (CSVD) is an umbrella term encompassing a spectrum of pathological processes affecting the cerebral small vessels, including arteriolosclerosis, small-vessel arteriosclerosis, cerebral amyloid angiopathy (CAA), microinfarcts, and lacunar infarcts. These pathological processes give rise to a range of neuroimaging manifestations, including white matter hyperintensities, lacunes, cerebral microbleeds, enlarged perivascular spaces, and brain atrophy. Although these entities are collectively classified as CSVD, the specific pathological subtypes and imaging phenotypes included under the CSVD designation vary across studies (Dupré et al., 2024; Kancheva et al., 2024; Pantoni, 2010; Wardlaw et al., 2013). Some studies identify CSVD primarily on the basis of one or several neuroimaging markers rather than comprehensive clinical characterization, and certain neuroimaging features are not entirely specific to CSVD and may also occur in other neurological conditions (Duering et al., 2023; Dupré et al., 2024; Kancheva et al., 2024). These differences in diagnostic approaches may contribute to heterogeneity across studies. CSVD is a major contributor to stroke, cognitive impairment, dementia, and gait disturbances, and its prevalence continues to increase with population aging (Blumen et al., 2023; Cannistraro et al., 2019; Wardlaw et al., 2022). As a syndrome, it is characterized by the coexistence of clinical manifestations, neuroimaging features, and pathological changes (Li et al., 2018; Ren et al., 2022). However, the underlying mechanisms driving the occurrence and progression of CSVD, as well as its associated cognitive impairment, remain poorly understood.

The glymphatic system (GS) is a cerebrospinal fluid–interstitial fluid exchange pathway mediated by aquaporin-4 that plays an important role in metabolic waste clearance from the brain (Nedergaard and Goldman, 2020; Plog and Nedergaard, 2018). In neurodegenerative diseases such as Alzheimer’s disease, dysfunction of glymphatic processes has been associated with ββ-amyloid accumulation and subsequent cognitive decline (Reeves et al., 2020). In CSVD, glymphatic processes may also be altered through multiple pathological and clinical factors. CSVD is pathologically characterized by arteriosclerosis, which contributes to reduced vascular pulsatility, a key driver of perivascular fluid transport. In addition, sleep disturbances, which are common in patients with CSVD, have also been associated with impaired glymphatic function. Together, these pathological and clinical factors may contribute to alterations in glymphatic processes in CSVD (Chong et al., 2022; Fu et al., 2006; Hablitz et al., 2020). Supporting this hypothesis, animal studies have demonstrated glymphatic dysfunction in CSVD models (Xue et al., 2020), whereas recent clinical studies have reported reduced the diffusion tensor image analysis along the perivascular space (DTI-ALPS) indices in patients with CSVD, which are closely associated with cognitive decline (Ai et al., 2025; Chen et al., 2025). Taken together, these findings suggest that alterations in glymphatic processes may represent one possible mechanism underlying CSVD-related cognitive impairment, although the specificity of the DTI-ALPS index as a measure of glymphatic function requires further investigation.

Given the difficulty of directly assessing glymphatic function in humans, increasing attention has been directed toward imaging-based surrogate markers. The DTI-ALPS index is a non-invasive DTI-based metric proposed to reflect glymphatic processes (Taoka et al., 2017, 2022, 2024). As an indirect imaging marker, the DTI-ALPS index reflects diffusion anisotropy in perivascular regions rather than direct glymphatic flow or clearance, and its biological specificity remains under active investigation (Kudo and Miki, 2026). Nevertheless, reduced DTI-ALPS indices have been reported in several neurological disorders associated with impaired perivascular clearance (Bae et al., 2021; Chen et al., 2021; Georgiopoulos et al., 2024; Si et al., 2022; Toh and Siow, 2021; Zhang et al., 2021). However, despite the growing number of studies, the association between the DTI-ALPS index and CSVD, particularly CSVD-associated cognitive impairment, has not yet been systematically synthesized. Therefore, the present study aimed to systematically evaluate alterations in the DTI-ALPS index in patients with CSVD and to examine its association with cognitive impairment.

## 2 Methods

This meta-analysis was conducted in accordance with the PRISMA 2020 statement (Page et al., 2021), and was prospectively registered in the PROSPERO database (registration number: CRD420261322688). The PRISMA 2020 checklist is provided in Supplementary Table 1.

### 2.1 Literature search

We systematically searched PubMed, Embase, the Cochrane Library, Web of Science, China National Knowledge Infrastructure (CNKI), Wanfang, SinoMed, and VIP databases from inception to February 1, 2026. The search focused on studies investigating the association between alterations in the DTI-ALPS index and cognitive impairment in cerebral small vessel disease. Search terms included “DTI-ALPS index,” “cerebral small vessel disease,” “cognitive impairment,” “glymphatic system,” and related terms. In addition, two reviewers (XXZ and MXX) independently screened the reference lists of all included studies and relevant reviews to identify any additional eligible studies not retrieved through the electronic database search. The detailed search strategy is provided in Supplementary Table 2.

### 2.2 Identification of eligible studies

Inclusion criteria

(1) studies reporting the DTI-ALPS index; (2) participants diagnosed with CSVD, including those with normal cognition, cognitive impairment, as well as healthy controls; (3) study designs including cross-sectional, case–control, or cohort studies; and (4) studies reporting the mean ± standard deviation of the DTI-ALPS index or providing data convertible to these measures.

Exclusion criteria

(1) duplicate publications, reviews, meta-analyses, and animal studies; (2) lack of full text or incomplete data; (3) non-Chinese or non-English publications; (4) studies involving participants with large-vessel disease or other neurological disorders; and (5) studies without an eligible comparison group relevant to the predefined meta-analysis comparisons.

Definitions of cognitive subgroups

For the purpose of this meta-analysis, CSVD-CI refers to patients with CSVD and cognitive impairment, including both mild cognitive impairment (MCI) and vascular dementia (VaD). CSVD-MCI refers to patients with CSVD and mild cognitive impairment, whereas CSVD-VaD refers to patients with CSVD and vascular dementia. CSVD-NC refers to patients with CSVD and normal cognition. Cognitive classifications were based on the diagnostic criteria adopted in the respective original studies.

### 2.3 Data extraction

Two reviewers (XXZ, MXX) independently extracted data using a standardized data extraction form. Discrepancies that could not be resolved through discussion were adjudicated by a fourth reviewer (XH). When available, the mean ± standard deviation (SD) of the DTI-ALPS index was directly extracted from the original articles. For studies presenting data only in graphical form without reported numerical values, data were extracted using WebPlotDigitizer (version 4.5). Prior to indirect data extraction, the authors were contacted via email up to three times to obtain the required data if available.

### 2.4 Quality assessment of literature

Two reviewers (XXZ and YYY) independently assessed the methodological quality of the included studies using the Newcastle-Ottawa Scale adapted for cross-sectional studies (NOS-xs) developed by Carra et al. (2025). The scale comprises six items with a maximum score of 9 stars, covering three domains: selection of study participants (2 stars), assessment of exposure and outcomes (4 stars), and control of confounding factors (3 stars). Studies with a total score of ≥7 stars were considered high quality, 4–6 stars as moderate quality, and ≤3 stars as low quality. Any discrepancies were resolved by consultation with a third reviewer (XH).

### 2.5 Statistical analysis

Statistical analyses were performed using Review Manager (version 5.4). As the DTI-ALPS index is a continuous variable, weighted mean differences (WMDs) with 95% confidence intervals (CIs) were used as pooled effect sizes. Heterogeneity was assessed using the Chi-square test (Cochran’s Q) and the I^2^ statistic (Higgins and Thompson, 2002). Random-effects models were used as the primary analyses for all comparisons because of expected clinical and methodological heterogeneity among studies. Sensitivity analyses were performed using fixed-effects models to evaluate the robustness of the pooled estimates. Forest plots were generated to present the pooled effect sizes, and *P* < 0.05 was considered statistically significant.

### 2.6 Subgroup analysis

Subgroup analyses were performed only for the primary comparison (CSVD vs. healthy controls) according to the CSVD subtype reported in the original studies, including sporadic CSVD, cerebral amyloid angiopathy (CAA), and white matter hyperintensity (WMH)-predominant CSVD. No subgroup analyses were conducted for the secondary comparisons because of the limited number of eligible studies. In this review, CSVD-VaD refers to patients with CSVD who were diagnosed with vascular dementia according to the diagnostic criteria adopted in the respective original studies.

### 2.7 Sensitivity analysis

For outcomes with significant heterogeneity, a leave-one-out sensitivity analysis was performed to assess the influence of each included study on the pooled results.

### 2.8 Publication bias

When the number of included studies was ≥10, funnel plots were generated and Egger’s regression test was performed using Stata (version 18.0) to assess publication bias (Egger et al., 1997).

## 3 Results

### 3.1 Literature search

**FIGURE 1:**
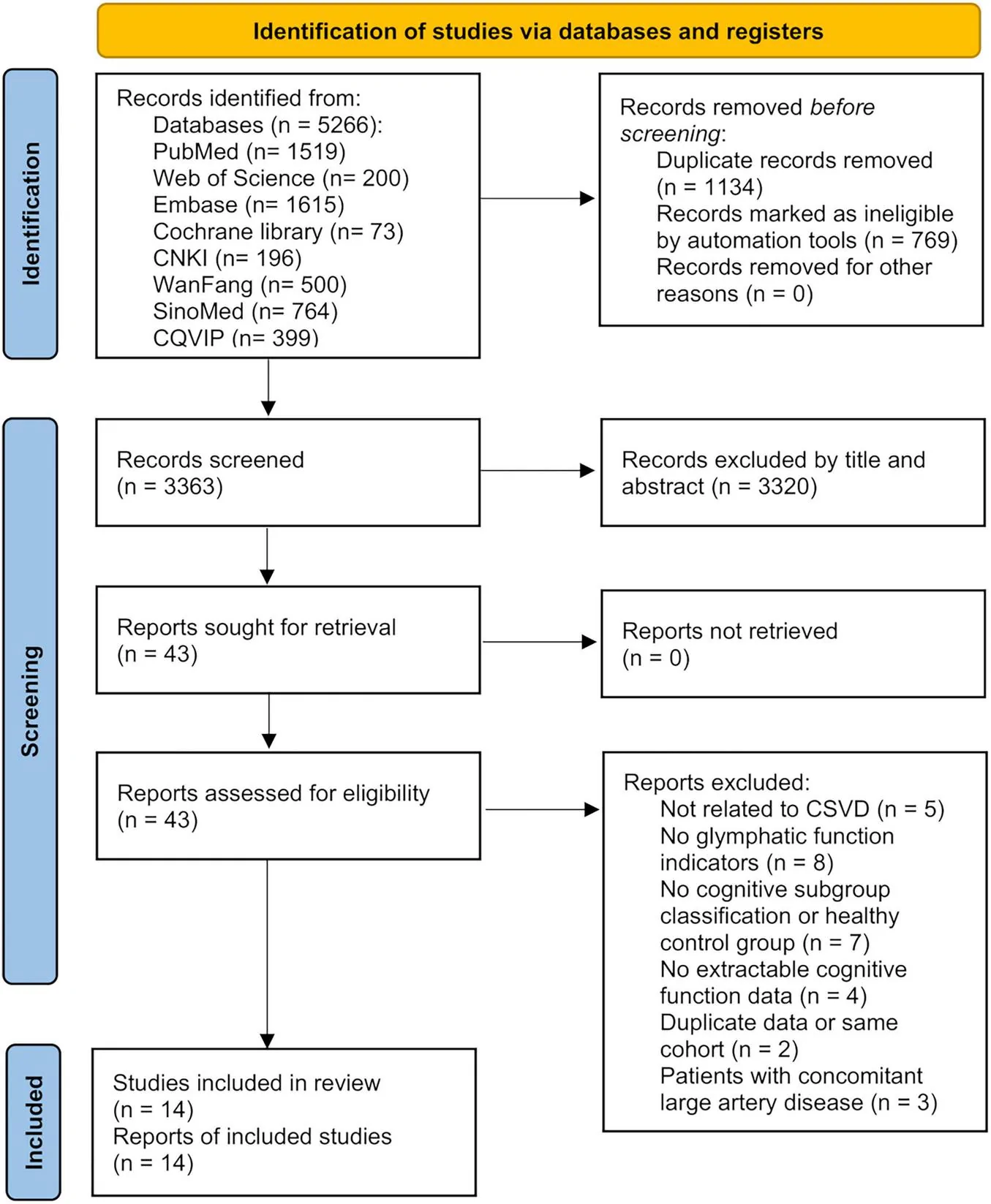
PRISMA 2020 flow diagram showing the study selection process.

A total of 5,266 records were initially identified from PubMed (*n* = 1,519), Web of Science (*n* = 200), Embase (*n* = 1,615), the Cochrane Library (*n* = 73), China National Knowledge Infrastructure (*n* = 196), Wanfang (*n* = 500), SinoMed (*n* = 764), and CQVIP (*n* = 399). After removing 1,134 duplicates and 769 records classified as reviews, meta-analyses, conference papers, or case reports, 3,363 records remained for screening. Following title and abstract screening, 3,320 records were excluded, leaving 43 articles for full-text assessment. Of these, 29 studies were excluded for reasons including irrelevance to CSVD (*n* = 5), lack of glymphatic function indicators (*n* = 8), absence of cognitive subgroup classification or healthy controls (*n* = 7), unavailable cognitive data (*n* = 4), duplicate or overlapping data (*n* = 2), and presence of large-vessel disease (*n* = 3). Ultimately, 14 studies met the inclusion criteria and were included in the meta-analysis (Ai et al., 2025; Chen et al., 2025; Huang et al., 2025; Ke et al., 2022; Lin et al., 2025; Lu et al., 2025; Qiu et al., 2025; Tang et al., 2022; Xu et al., 2022, 2024, 2025; Zeng et al., 2025; Zhao et al., 2024, 2025), as illustrated in Figure 1.

### 3.2 Study characteristics

A total of 14 studies published between 2022 and 2026 were included, comprising 2,149 participants, including 620 healthy controls and 1,529 patients with CSVD (530 with cognitive impairment and 408 cognitively normal). The main characteristics of the included studies are summarized in Table 1. All studies were conducted in China and employed a cross-sectional design. Although several studies were conducted at the same institutions or in the same geographic regions, we assessed cohort overlap by comparing sample sizes, recruitment periods, and inclusion/exclusion criteria. Studies that explicitly reported overlapping data (*n* = 2) were excluded. For the remaining 14 studies, we confirmed that the cohorts were distinct based on recruitment periods, inclusion criteria, and sample characteristics. A detailed summary of study sources is provided in Supplementary Table 3. Regarding comparisons involving the DTI-ALPS index, 12 studies compared patients with CSVD and healthy controls. Eight studies compared CSVD with cognitive impairment (CSVD-CI) with cognitively normal CSVD (CSVD-NC), five compared CSVD with mild cognitive impairment (CSVD-MCI) with CSVD-NC, and three compared CSVD with vascular dementia (CSVD-VaD) with CSVD-NC. In addition, three studies compared CSVD-VaD with CSVD-MCI. In terms of MRI parameters, all studies used a field strength of 3.0 T, except for one study that used 7.0 T. All studies employed a b-value of 1000 s/mm^2^. Diffusion directions and coil configurations varied across studies; among those reporting these parameters, the most commonly used numbers of diffusion directions were 64 and 20, and the most frequently used coil type was a 32-channel head coil. Cognitive function was assessed using instruments with varying levels of specificity. Most studies relied on global cognitive screening tools, primarily the Mini-Mental State Examination (MMSE) and the Montreal Cognitive Assessment (MoCA). Several studies additionally incorporated domain-specific neuropsychological tests covering multiple domains, including memory (e.g., AVLT, RAVLT, WMS), executive function (e.g., TMT, Stroop, VFT), attention and processing speed (e.g., SDMT, DSST), language (e.g., BNT), and visuospatial ability (e.g., RCFT, CDT). However, the selection and reporting of these tests varied considerably across studies. A small number of studies used clinical rating scales such as the Clinical Dementia Rating (CDR) for cognitive classification rather than quantitative assessment. Overall, this heterogeneity limited the comparability of domain-specific outcomes. The median NOS-xs score of the included studies was 8 (range: 6–9, Table 2). Only one study scored 6, while all others scored ≥7. According to the predefined NOS-xs criteria, most studies were classified as high quality, and no studies were considered low quality. However, NOS-xs scores primarily reflect general study design and reporting quality and may not fully capture DTI-ALPS-specific methodological variability. The diagnostic criteria used to define CSVD varied across studies (Supplementary Table 4). Most studies diagnosed sporadic CSVD according to the STRIVE or STRIVE-2 criteria, whereas CAA and WMH-predominant CSVD were identified using the Boston criteria and MRI-defined WMH burden, respectively.

**TABLE 1:** Characterization of the studies included in the systematic review.

Author, Year	Region	Study design	Field strength	Coil	Directions	b-value (s/mm^2^)	CSVD group					
							CSVD Subtype	Age (years)	Male (%)	Sample size	Cognitive assessment	DTI-ALPS index
Xu et al., 2022	China	Cross-sectional	3.0T	NR	NR	1000	CAA	69 (60–73)*	39 (61.9)	63	MMSE, MoCA	CSVD: 1.216 ± 0.122
Tang et al., 2022	China	Cross-sectional	3.0T	8-ch	20	1000	Sporadic CSVD	65.5 ± 7.7	85 (63.9)	133	MoCA; AVLT, TMT-A/B, VFT, SDMT, BNT, RCFT	CSVD-NC: 1.054 ± 0.142; CSVD-CI: 0.958 ± 0.088
Qiu et al., 2025	China	Cross-sectional	3.0T	8-ch	20	1000	Sporadic CSVD	64.8 ± 6.0	82 (80.4)	102	MMSE, MoCA; TMT-A/B, Stroop, RCFT, AVLT, BNT, VFT	CSVD: 1.244 ± 0.168
Xu et al., 2024	China	Cross-sectional	3.0T	12-ch	30	1000	WMH	68.4 ± 7.7	85 (41.3)	206	MMSE; AVLT, Logical Memory, RCFT, CDT, DSST, TMT-A/B, Stroop, VFT, DST, Similarity	CSVD: 1.333 ± 0.151
Zeng et al., 2025	China	Cross-sectional	3.0T	21/48-ch	20	1000	WMH	53.0 ± 3.7	34 (40.5)	84	MMSE; DST, DSST, BNT	CSVD: 1.426 ± 0.192
Zhao et al., 2025	China	Cross-sectional	3.0T	32-ch	64	1000	Sporadic CSVD	68 (64–72)*	45 (55.6)	81	MMSE, MoCA	CSVD-NC: 1.418 (1.250–1.545)*; CSVD-MCI: 1.313 (1.196–1.421)*; CSVD-VaD: 1.280 ± 0.100
Ai et al., 2025	China	Cross-sectional	3.0T	24-ch	64	1000	Sporadic CSVD	63.7 ± 6.8	61 (50.8)	120	CDR	CSVD-NC: 1.29 ± 0.11; CSVD-MCI: 1.23 ± 0.11
Xu et al., 2025	China	Cross-sectional	3.0T	NR	64	1000	Sporadic CSVD	69.2 ± 6.4	86 (53.8)	160	MMSE, MoCA; TMT-A/B, Stroop, DST, AVLT, CDT	CSVD-NC: 1.41 ± 0.13; CSVD-MCI: 1.35 ± 0.14
Huang et al., 2025	China	Cross-sectional	3.0T	NR	34	1000	Sporadic CSVD	68.4 ± 6.4	25 (34.7)	72	MMSE, MoCA	CSVD: 1.225 ± 0.139
Zhong et al., 2024	China	Cross-sectional	3.0T	16-ch	20	1000	Sporadic CSVD	67.2 ± 6.1	37 (52.8)	70	MoCA	CSVD-NC: 1.428 ± 0.142; CSVD-CI: 1.282 ± 0.194
Lu et al., 2025	China	Cross-sectional	3.0T	19-ch	NR	1000	Sporadic CSVD	63 (56–70)*	62 (60.8)	102	MoCA	CSVD-NC: 1.41 ± 0.17; CSVD-MCI: 1.34 ± 0.16; CSVD-VaD: 1.33 ± 0.17
Lin et al., 2025	China	Cross-sectional	7.0T	32-ch	NR	1000	CAA	62.4 ± 9.7	39 (60.9)	64	MMSE, MoCA; RAVLT, BNT, TMT-A, Stroop, DST	CSVD: 1.31 ± 0.11
**Chen et al., 2025**	China	Cross-sectional	3.0T	32-ch	64	1000	Sporadic CSVD	68.2 ± 6.2	55 (40.7)	135	MMSE, MoCA	NR
Ke et al., 2022	China	Cross-sectional	3.0T	32-ch	32	1000	Sporadic CSVD	66.7 ± 8.2	68 (50.0)	137	MMSE, MoCA, CDR; AVLT, BNT, VFT, TMT-A/B, Stroop, CDT, WMS	CSVD-NC: 1.64 ± 0.20; CSVD-MCI: 1.52 ± 0.20; CSVD-VaD: 1.44 ± 0.26
**Author**			**HC group**									
			**Age**			**Male**			**Sample**		**ALPS index**	
Qiu et al., 2025			62.45 ± 6.69			22 (75.9%)			29		1.345 ± 0.176	
Xu et al., 2025			62.43 ± 7.28			75 (39.5%)			190		1.48 ± 0.13	
Huang et al., 2025			58.90 ± 6.67			15 (37.5%)			40		1.29 ± 0.13	
Ke et al., 2022			58.88 ± 6.41			27 (51.92%)			52		1.76 ± 0.18	
Xu et al., 2024			61.6 ± 7.8			13 (30.2%)			43		1.39 ± 0.16	
Xu et al., 2022			65 (62, 70)*			36 (51.4%)			70		1.319 ± 0.140	
Ai et al., 2025			60.18 ± 4.57			19 (47.50%)			40		1.39 ± 0.14	
Lu et al., 2025			59 (57, 63)*			12 (41.4%)			29		1.50 ± 0.19	
Zhao et al., 2025			65 (61, 68)*			9 (45.0%)			20		1.540(1.452, 1.667)*	
Zhong et al., 2024			64.70 ± 4.76			15 (40.5%)			37		1.532 ± 0.175	
Zeng et al., 2025			52.30 ± 3.71			8 (34.8%)			23		1.52 ± 0.16	
Lin et al., 2025			62.85 ± 5.67			28 (59.6%)			47		1.47 ± 0.11	

*Median[range]; CSVD, cerebral small vessel disease; HC, healthy control; CAA, cerebral amyloid angiopathy; WMH, white matter hyperintensity; NC, normal cognition; CI, cognitive impairment; MCI, mild cognitive impairment; VaD, vascular dementia; NR, not reported; MMSE, Mini-Mental State Examination; MoCA, Montreal Cognitive Assessment; CDR, Clinical Dementia Rating; TMT, Trail Making Test; AVLT, Auditory Verbal Learning Test; VFT, Verbal Fluency Test; SDMT, Symbol Digit Modalities Test; BNT, Boston Naming Test; RCFT, Rey-Osterrieth Complex Figure Test; DST, Digit Span Test; DSST, Digit Symbol Substitution Test; CDT, Clock Drawing Test; WMS, Wechsler Memory Scale; HC, healthy control.

**TABLE 2:** Risk of bias assessment according to the Newcastle–Ottawa Scale for cross-sectional studies.

References	Study design	Selection	Exposure/Outcome	Confounding factors	Total
Xu et al., 2022	Cross-sectional study	*	****	***	8
Tang et al., 2022	Cross-sectional study	*	****	***	8
Qiu et al., 2025	Cross-sectional study	*	****	***	8
Xu et al., 2024	Cross-sectional study	*	****	***	8
Zeng et al., 2025	Cross-sectional study	*	****	***	8
Zhao et al., 2025	Cross-sectional study	*	****	**	7
Ai et al., 2025	Cross-sectional study	*	****	***	8
Xu et al., 2025	Cross-sectional study	*	****	***	8
Huang et al., 2025	Cross-sectional study	*	****	*	6
Zhong et al., 2024	Cross-sectional study	*	****	***	8
Lu et al., 2025	Cross-sectional study	*	****	***	8
Lin et al., 2025	Cross-sectional study	*	****	***	8
Chen et al., 2025	Cross-sectional study	**	****	***	9
Ke et al., 2022	Cross-sectional study	*	****	***	8

****4 points; ***3 points; **2 points; *1 point.

### 3.3 The results of meta-analysis

#### 3.3.1 DTI-ALPS index in CSVD vs. healthy controls

**FIGURE 2:**
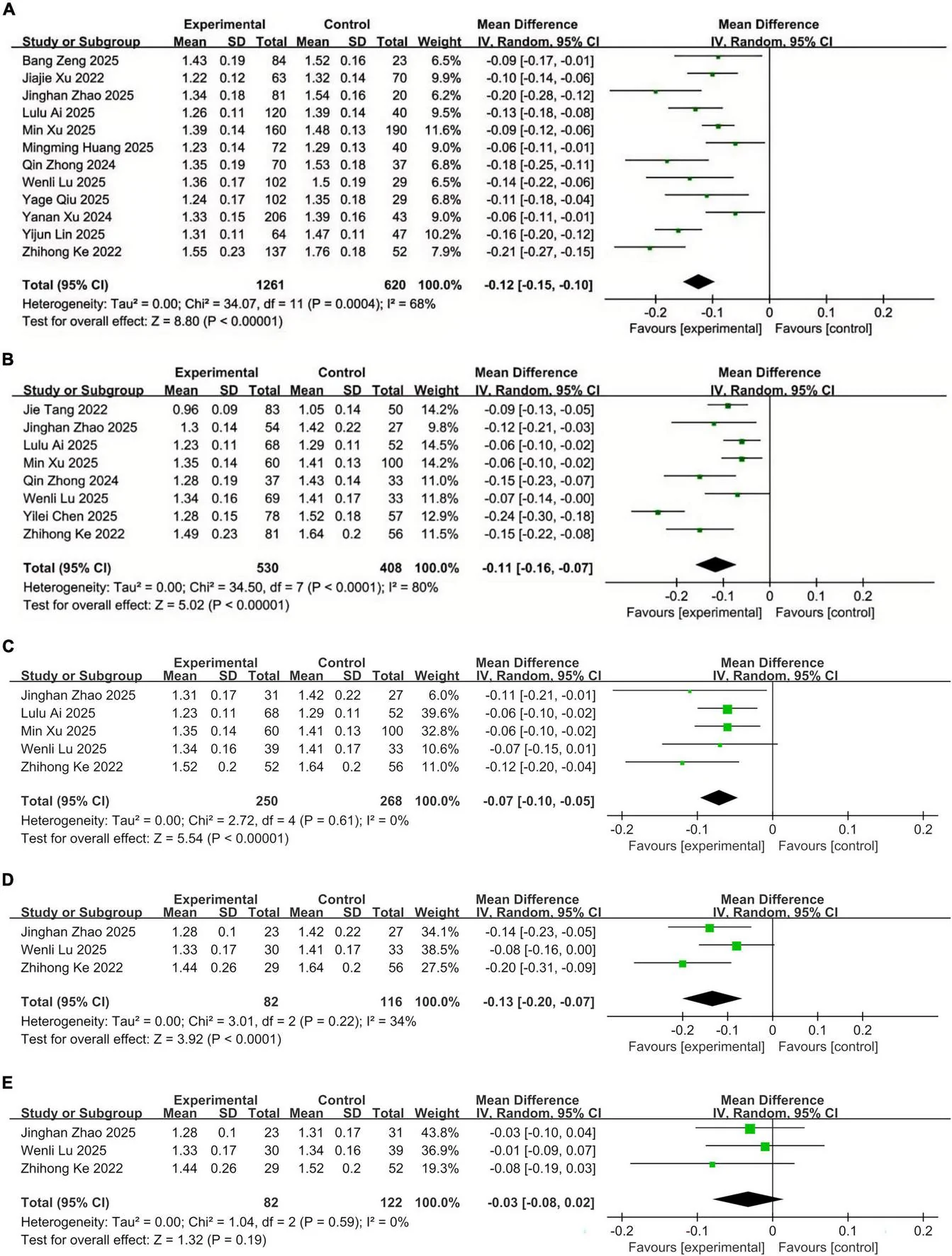
Forest plots of the diffusion tensor imaging analysis along the perivascular space (DTI-ALPS) index across different comparisons: **(A)** cerebral small vessel disease (CSVD) vs. healthy controls (HC); **(B)** CSVD with cognitive impairment (CSVD-CI) vs. CSVD with normal cognition (CSVD-NC); **(C)** CSVD with mild cognitive impairment (CSVD-MCI) vs. CSVD-NC; **(D)** CSVD with vascular dementia (CSVD-VaD) vs. CSVD-NC; **(E)** CSVD-VaD vs. CSVD-MCI.

A total of 12 studies compared the DTI-ALPS index between patients with cerebral small vessel disease (CSVD) and healthy controls (HC). A random-effects meta-analysis showed that the DTI-ALPS index was significantly lower in the CSVD group than in the HC group (*P* < 0.00001), with a pooled WMD of -0.12 (95% CI: -0.15 to -0.10). Moderate heterogeneity was observed (I^2^ = 68%, *P* = 0.0004) (Figure 2A). The corresponding 95% prediction interval ranged from -0.22 to -0.03, indicating that future studies are expected to show a consistent direction of association.

#### 3.3.2 DTI-ALPS index in CSVD-CI vs. CSVD-NC

Eight studies compared the DTI-ALPS index between the CSVD with cognitive impairment (CSVD-CI) group and the CSVD with normal cognition (CSVD-NC) group. A random-effects meta-analysis showed that the DTI-ALPS index was significantly lower in the CSVD-CI group than in the CSVD-NC group (*P* < 0.00001), with a pooled WMD of -0.11 (95% CI: -0.16 to -0.07). High heterogeneity was observed (I^2^ = 80%, *P* < 0.0001) (Figure 2B).

#### 3.3.3 DTI-ALPS index in CSVD-MCI vs. CSVD-NC

Five studies compared the DTI-ALPS index between the CSVD with mild cognitive impairment (CSVD-MCI) group and the CSVD with normal cognition (CSVD-NC) group. A random-effects meta-analysis showed that the DTI-ALPS index was significantly lower in the CSVD-MCI group than in the CSVD-NC group (*P* < 0.00001), with a pooled WMD of -0.07 (95% CI: -0.10 to -0.05). No heterogeneity was observed (I^2^ = 0%, *P* = 0.61) (Figure 2C).

#### 3.3.4 DTI-ALPS index in CSVD-VaD vs. CSVD-NC

Three studies compared the DTI-ALPS index between the CSVD with vascular dementia (CSVD-VaD) group and the CSVD with normal cognition (CSVD-NC) group. A random-effects meta-analysis showed that the DTI-ALPS index was significantly lower in the CSVD-VaD group than in the CSVD-NC group (*P* < 0.00001), with a pooled WMD of -0.13 (95% CI: -0.20 to -0.07). No significant heterogeneity was observed (I^2^ = 34%, *P* = 0.22) (Figure 2D).

#### 3.3.5 DTI-ALPS index in CSVD-VaD vs. CSVD-MCI

Three studies compared the DTI-ALPS index between the CSVD with vascular dementia (CSVD-VaD) group and the CSVD with mild cognitive impairment (CSVD-MCI) group. A random-effects meta-analysis showed no significant difference in the DTI-ALPS index between the two groups (*P* = 0.19), with a pooled WMD of -0.03 (95% CI: -0.08 to 0.02). No heterogeneity was observed (I^2^ = 0%, *P* = 0.59) (Figure 2E).

### 3.4 Subgroup analysis

**FIGURE 3:**
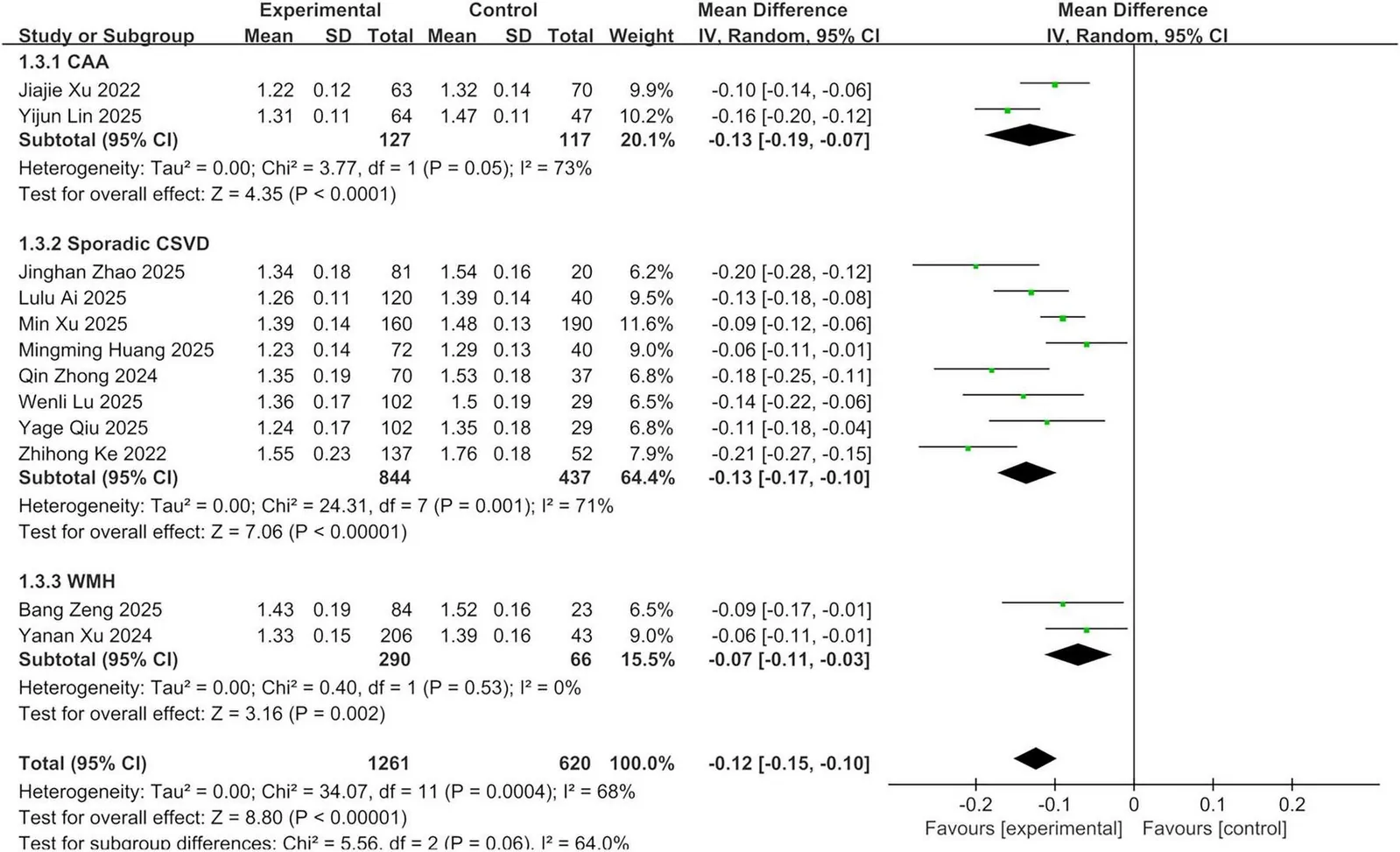
Forest plots of subgroup analyses of the DTI-ALPS index for CSVD vs. HC.

The pooled results of the three subgroup meta-analyses showed that the cerebral amyloid angiopathy (CAA) subgroup (WMD = -0.13, 95% CI: -0.19 to -0.07, I^2^ = 73%, *P* = 0.05), the sporadic CSVD subgroup (WMD = -0.13, 95% CI: -0.17 to -0.10, I^2^ = 71%, *P* = 0.001), and the white matter hyperintensity (WMH) subgroup (WMD = -0.07; 95% CI: -0.11 to -0.03; I^2^ = 0%, *P* = 0.53) all showed significantly lower DTI-ALPS indices compared with healthy controls (all overall *P* < 0.01). Similar effect estimates were observed in the CAA and sporadic CSVD subgroups (-0.13), while the WMH subgroup showed a numerically smaller effect estimate (-0.07). Subgroup difference testing indicated that the differences in effect sizes among subtypes did not reach statistical significance (χ^2^ = 5.56, *P* = 0.06). Notably, substantial heterogeneity was observed in the CAA and sporadic CSVD subgroups (I^2^ = 73% and 71%, respectively), whereas no heterogeneity was detected in the WMH subgroup (I^2^ = 0%) (Figure 3).

### 3.5 Sensitivity analysis

**FIGURE 4:**
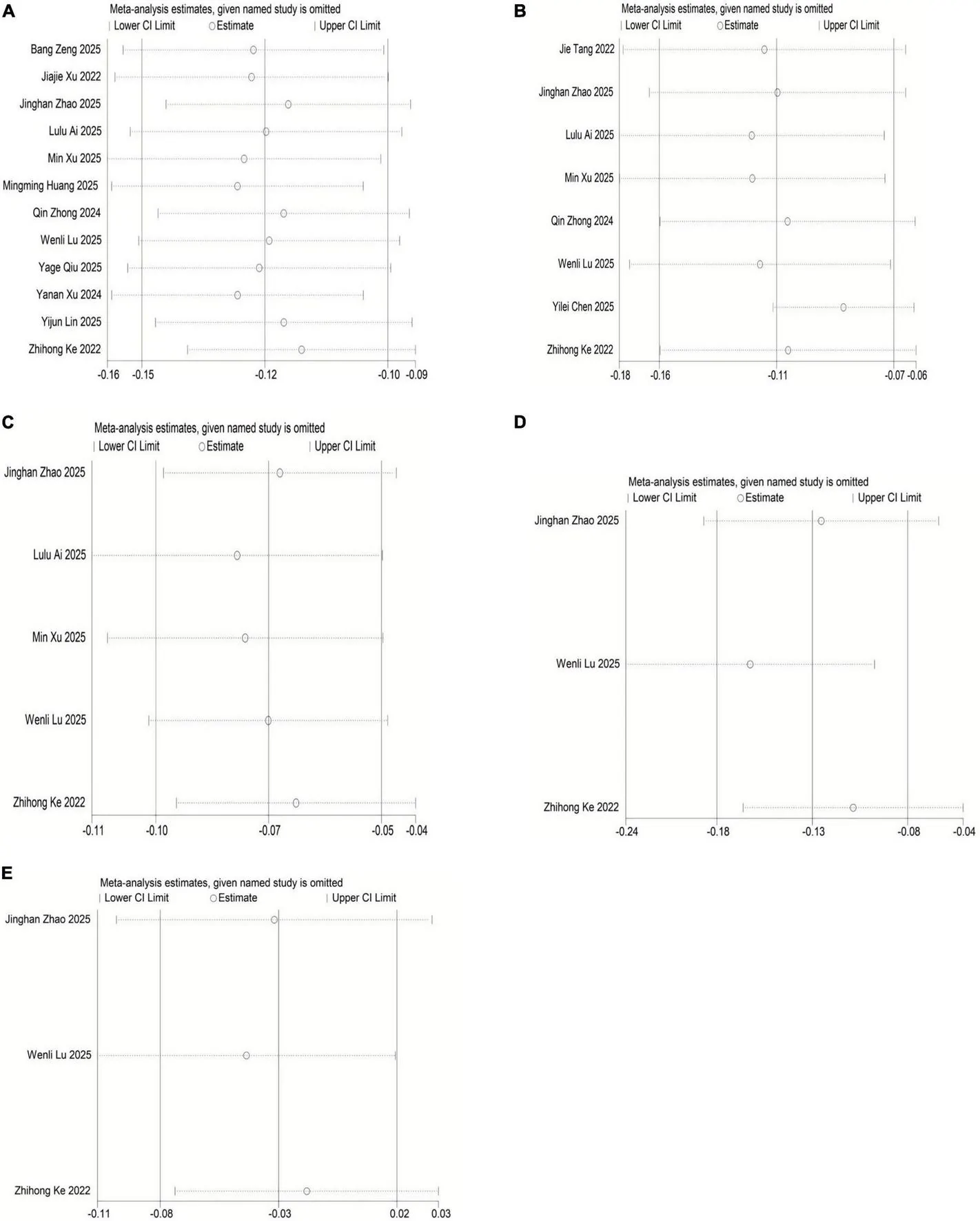
Sensitivity analysis of the DTI-ALPS index across different comparisons: **(A)** CSVD vs. HC; **(B)** CSVD-CI vs. CSVD-NC; **(C)** CSVD-MCI vs. CSVD-NC; **(D)** CSVD-VaD vs. CSVD-NC; **(E)** CSVD-VaD vs. CSVD-MCI.

A leave-one-out sensitivity analysis was conducted for the five primary comparisons to assess the influence of each individual study on the pooled WMD. Except for the comparison between the CSVD-CI and CSVD-NC groups (Figure 4B), where exclusion of the study by Chen et al. (2025) markedly reduced heterogeneity from 80.0% to 36.0% and changed the pooled WMD from -0.115 to -0.089, the pooled effect sizes in the remaining comparisons (Figures 4A, C–E) did not change substantially after removing any single study.

Sensitivity analyses using fixed-effects models were performed for comparisons with low statistical heterogeneity (I^2^ < 50%) to assess the robustness of the primary random-effects analyses. The pooled effect estimates remained comparable between random-effects and fixed-effects models, with no meaningful changes in effect direction or statistical interpretation observed (Supplementary Table 5).

### 3.6 Publication bias

**FIGURE 5:**
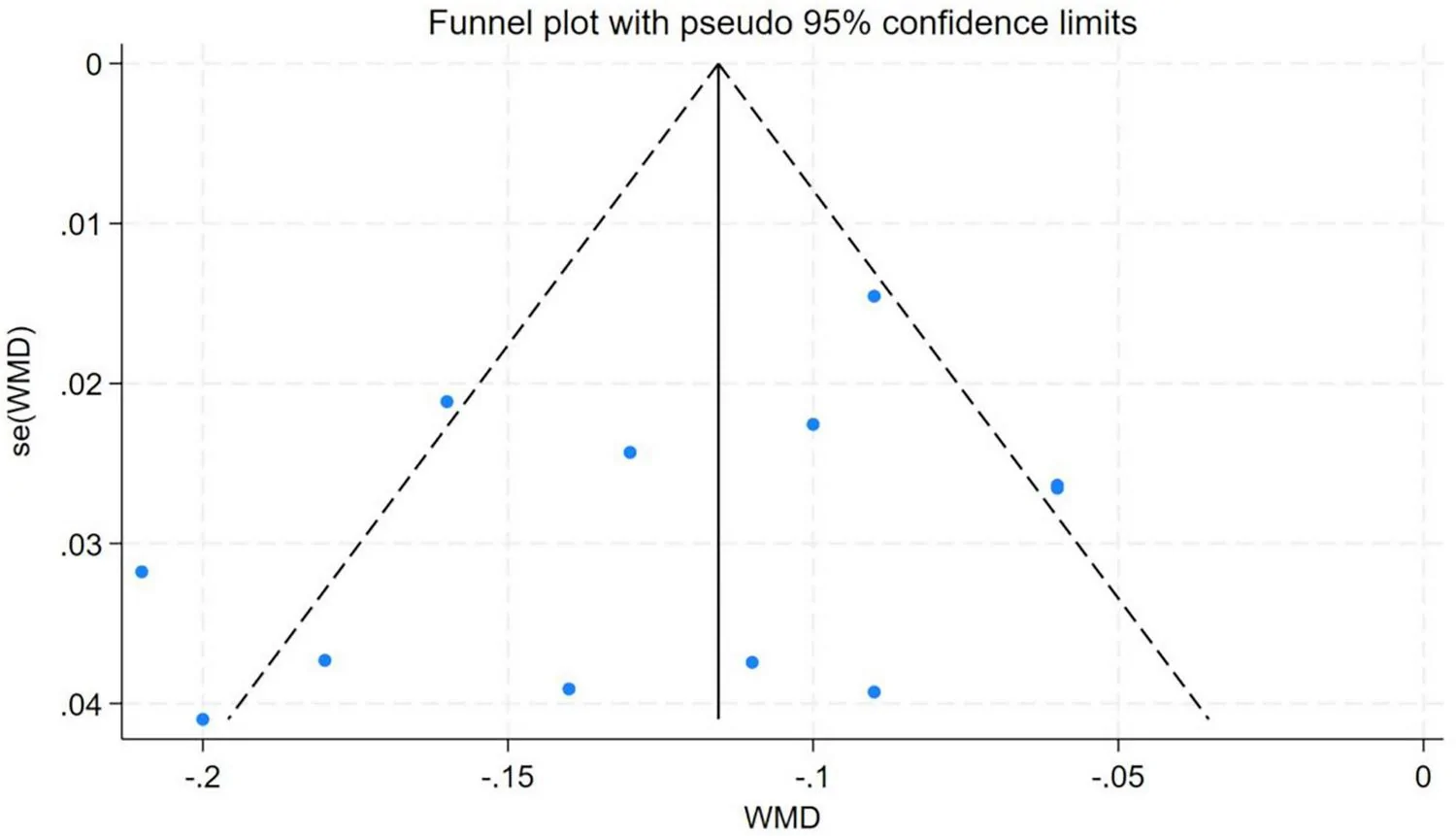
Funnel plot of publication bias for the DTI-ALPS index in CSVD vs. HC.

We assessed potential publication bias in the comparison of the DTI-ALPS index between CSVD and HC. Both Egger’s test (*P* = 0.189) and visual inspection of the funnel plot indicated no evidence of publication bias (Figure 5).

## 4 Discussion

In this meta-analysis, the DTI-ALPS index was significantly reduced in patients with CSVD compared with healthy controls. Within the CSVD population, patients with cognitive impairment (CSVD-CI) showed lower DTI-ALPS indices than cognitively normal individuals (CSVD-NC). Notably, a significant reduction was already evident at the CSVD-MCI stage, whereas no further significant decline was observed in CSVD-VaD. These findings indicate that lower DTI-ALPS indices are associated with the presence of cognitive impairment in CSVD, but the available cross-sectional data do not permit conclusions about whether this association reflects a temporal sequence or can be used for staging. Whether the DTI-ALPS index can serve as a marker for early detection or disease staging requires further investigation in longitudinal studies.

The GS plays a central role in maintaining brain homeostasis through metabolic waste clearance, fluid exchange, and immune regulation (Astara et al., 2023; Iliff et al., 2012). Its impairment may lead to the accumulation of neurotoxic metabolites, triggering neuroinflammation and neuronal injury, thereby contributing to cognitive decline (Lee et al., 2024; Xie et al., 2013; Zou et al., 2024). Under physiological conditions, cerebrospinal fluid flows along periarterial spaces, exchanges with interstitial fluid, and is cleared via perivenous pathways, a process largely driven by arterial pulsatility (Kedarasetti et al., 2020; Lee et al., 2024; Shi et al., 2020). In CSVD, small vessel pathology reduces arterial pulsatility and may disrupt this process (Cao et al., 2025; Moretti and Caruso, 2020; Wardlaw et al., 2020). These mechanisms provide a plausible link between CSVD pathology and cognitive impairment. Importantly, the DTI-ALPS index, as a non-invasive marker proposed to reflect GS-related processes, has been increasingly investigated for its association with CSVD and related cognitive decline.

Evidence from other neurological conditions further supports the association between the DTI-ALPS index and cognitive impairment. A meta-analysis by Khalafi et al., (2026) demonstrated that patients with Alzheimer’s disease exhibit lower DTI-ALPS indices than healthy controls, with lower DTI-ALPS indices observed across different cognitive states from subjective cognitive decline (SCD) to MCI and Alzheimer’s disease. Ma et al. (2021) reported that the DTI-ALPS index was significantly lower in Parkinson’s disease patients with cognitive impairment compared to cognitively normal individuals, suggesting an association between DTI-ALPS alterations and cognitive impairment in Parkinson’s disease. Ghaderi et al. (2025) further showed through meta-analysis that patients with MCI have reduced DTI-ALPS indices compared with healthy controls, highlighting its potential as an imaging metric associated with cognitive decline and warranting further investigation. Reduced DTI-ALPS indices were observed across different cognitive states in CSVD (Tang et al., 2022). Consistent with prior findings, our results showed that CSVD patients across different cognitive states exhibit reduced DTI-ALPS indices compared with healthy controls, with a numerical trend toward lower DTI-ALPS indices across cognitive groups. However, no significant difference was observed between the MCI and VaD groups (Ke et al., 2022; Lu et al., 2025; Zhao et al., 2025). This finding should be interpreted with caution and does not establish the value of the DTI-ALPS index for early detection or disease staging. Rather, it may reflect the limited number of available studies, heterogeneous diagnostic criteria, insufficient statistical power, potential ceiling effects, or methodological limitations of the DTI-ALPS index itself. Therefore, the current evidence supports an association between lower DTI-ALPS indices and the presence of cognitive impairment, whereas its value for disease staging remains uncertain and requires confirmation in future longitudinal studies.

Subgroup analyses indicated numerical differences in alterations of the DTI-ALPS index across CSVD subtypes. Differences in effect estimates between the sporadic CSVD and WMH subgroups suggest numerical differences in the magnitude of DTI-ALPS reduction among subtypes. WMH is a common neuroimaging manifestation of CSVD (Sperber et al., 2023), with a numerically smaller reduction in the DTI-ALPS index, whereas a greater reduction in the DTI-ALPS index was observed in the sporadic CSVD subgroup. However, these subgroup findings should be interpreted cautiously and regarded as exploratory because of the limited number of studies and potential differences in participant characteristics, imaging protocols, and diagnostic criteria across subgroups. Whether alterations in the DTI-ALPS index differ according to CSVD subtype requires further investigation in larger, well-characterized cohorts. These findings should be considered hypothesis-generating and require further validation before the potential clinical utility of the DTI-ALPS index can be determined.

Another potential source of clinical heterogeneity is the variation in diagnostic criteria used to define CSVD across the included studies. As summarized in Supplementary Table 4, although most studies diagnosed sporadic CSVD according to the STRIVE or STRIVE-2 criteria, additional inclusion requirements varied considerably, including differences in Fazekas score thresholds, total CSVD burden scores, and the required combination of neuroimaging markers such as lacunes, cerebral microbleeds, or enlarged perivascular spaces. Furthermore, studies focusing on specific CSVD phenotypes applied different diagnostic frameworks, with cerebral amyloid angiopathy defined using the Modified Boston criteria or Boston criteria version 2.0, and WMH-predominant CSVD identified primarily based on white matter hyperintensity burden. These differences likely reflect variations in disease spectrum and severity across study populations and may have contributed to the between-study heterogeneity observed in this meta-analysis.

Several methodological factors related to MRI acquisition and DTI-ALPS processing may also have contributed to the heterogeneity observed across studies (Supplementary Table 6). First, preprocessing pipelines varied, with most studies using FSL-based tools, whereas others employed MRtrix3 or DSI Studio. Second, tensor fitting methods differed across studies. Third, ROI placement was performed manually in most studies, while some studies used automated or semi-automated atlas-based approaches. Fourth, the DTI-ALPS index was calculated either unilaterally (left hemisphere only) or bilaterally (using averaged values), and ROI sizes ranged from 2.5 mm to 6 mm. These methodological differences, summarized in Supplementary Table 6, may influence DTI-ALPS measurements independently of glymphatic processes and should therefore be considered when interpreting the pooled findings.

Sensitivity analysis showed that exclusion of the study by Chen et al., (2025) markedly reduced heterogeneity in the comparison between CSVD-CI and CSVD-NC, indicating that this study was a major source of variability. This may be related to its inclusion of neurovascular coupling metrics, which could influence the relationship between DTI-ALPS and cognition. Nevertheless, after exclusion of this study, heterogeneity was substantially reduced and the pooled effect remained statistically significant (WMD changed from -0.115 to -0.089), supporting the stability of the overall association. However, the magnitude of the pooled effect should be interpreted with appropriate caution. One study (Lin et al., 2025) used 7.0T MRI while the remainder used 3.0T; we did not exclude this study *a priori*, but we note this as a potential source of methodological heterogeneity. A sensitivity analysis excluding this study showed no substantial change in the pooled estimate (see Figure 4A).

Although the sensitivity analyses supported the robustness of the pooled estimates, additional sources of heterogeneity should also be considered. Global screening tools such as the MMSE and MoCA were most commonly used, these measures provide only a general evaluation of cognitive status and are relatively insensitive to domain-specific impairments. Several studies employed more comprehensive neuropsychological batteries assessing multiple domains, including memory, executive function, attention, language, and visuospatial ability, suggesting that alterations in the DTI-ALPS index in CSVD may involve multi-domain cognitive deficits. However, substantial variability in test selection, scoring, and reporting limited the feasibility of quantitative synthesis of domain-specific outcomes. In addition, some studies relied on clinical classification tools such as the CDR, which may further reduce sensitivity for detecting subtle cognitive changes. Consequently, this meta-analysis primarily focused on global cognitive impairment, and domain-specific associations remain to be clarified. This limitation is consistent with our preregistered protocol, which initially aimed to assess domain-specific outcomes but could not be fully implemented due to the lack of standardized data. Future studies using harmonized neuropsychological batteries are needed to better characterize these relationships. Beyond differences in cognitive assessment, the interpretation of the DTI-ALPS index itself also warrants careful consideration.

Several important methodological considerations should be taken into account when interpreting the DTI-ALPS index as a surrogate imaging marker of glymphatic processes. Although the DTI-ALPS index has been proposed to reflect glymphatic function, its biological specificity remains under active investigation. The ALPS index is derived from a periventricular white matter region where projection and association fibers intersect, making it susceptible to crossing-fiber geometry and partial volume effects (Taoka et al., 2024). Consistent with this concern, Georgiopoulos et al. (2024) demonstrated that ALPS measurements may be substantially influenced by fiber orientation independent of perivascular fluid transport, suggesting that the index may partly reflect underlying white matter microstructural characteristics rather than glymphatic processes alone. Furthermore, Mossige et al. (2026) recently compared the DTI-ALPS index with intrathecal contrast-enhanced MRI, a more direct imaging approach for assessing glymphatic transport, and reported only limited correspondence between the two techniques, further indicating that the DTI-ALPS index should be regarded as an indirect rather than definitive measure of glymphatic function. Acquisition parameters may also contribute to measurement variability. Vuong et al. (2026) reported that the commonly used b-value of 1000 s/mm^2^ may not be optimal for characterizing diffusion along perivascular spaces, a finding that is particularly relevant because all studies included in the present meta-analysis employed this acquisition parameter. In addition, the DTI-ALPS index may be influenced by several disease- and anatomy-related factors, including ventricular enlargement, brain atrophy, white matter lesion burden, enlarged perivascular spaces, MRI acquisition protocols, preprocessing strategies, and ROI placement, all of which are common in patients with CSVD and may confound ALPS measurements independently of glymphatic alterations. Therefore, although our meta-analysis consistently demonstrated lower DTI-ALPS indices in patients with CSVD and cognitive impairment, these findings should be interpreted as evidence of an association between altered DTI-ALPS measurements and disease status rather than definitive evidence of impaired glymphatic function. Nevertheless, the consistent reduction in DTI-ALPS indices observed across independent studies suggests that this diffusion-based metric remains a promising imaging biomarker for characterizing CSVD and its associated cognitive impairment. Future methodological studies directly comparing DTI-ALPS with complementary imaging modalities and fluid-based biomarkers are warranted to further clarify its biological specificity and potential clinical utility.

Several limitations should be acknowledged. First, because all included studies were cross-sectional, the observed associations should not be interpreted as evidence of temporal or causal relationships between alterations in the DTI-ALPS index and cognitive impairment. Longitudinal studies are needed to determine whether changes in DTI-ALPS precede, accompany, or follow cognitive decline in patients with CSVD. Second, all pooled analyses were based on unadjusted group means, as the included studies did not consistently report adjusted estimates for the DTI-ALPS index. Consequently, the observed associations may be confounded by age, sex, brain atrophy, ventricular size, scanner differences, and CSVD severity. Future individual-level data meta-analyses are needed to account for these covariates. Third, cognitive assessment varied across studies. Although MMSE and MoCA were the most commonly used screening tools, differences in neuropsychological batteries and cognitive classification criteria limited the quantitative synthesis of domain-specific outcomes. Therefore, the present meta-analysis primarily evaluated global cognitive impairment, and no firm conclusions can be drawn regarding specific cognitive domains or cognitive severity. Fourth, methodological heterogeneity should be considered. Differences in MRI acquisition parameters (e.g., field strength, head coil, diffusion directions) and DTI-ALPS processing methods (Supplementary Table 6) may have influenced the pooled estimates. Although the primary comparison included 12 studies, meta-regression was not performed because key clinical covariates and adjusted estimates were inconsistently reported across studies, and imaging-related variables showed limited variability or highly unbalanced distributions, precluding reliable estimation. Fifth, all studies were conducted in China, which may limit generalizability. Finally, publication bias was formally assessed only for the comparison including at least ten studies (CSVD vs. HC, *n* = 12), consistent with the recommendations of the Cochrane Handbook. Egger’s test did not indicate significant publication bias (*P* = 0.189). For the remaining comparisons, formal assessment of publication bias was not appropriate because fewer than ten studies were available; therefore, publication bias cannot be excluded.

Despite these limitations, the findings provide potential clinical implications. The DTI-ALPS index may serve as a non-invasive imaging metric associated with cognitive impairment in CSVD. The significant reduction in DTI-ALPS indices observed in patients with CSVD-MCI suggests that this diffusion-based metric may provide imaging evidence associated with cognitive impairment in patients with CSVD. However, given the cross-sectional nature of the available evidence and the absence of significant differences between the CSVD-MCI and CSVD-VaD groups, its value for early detection, disease staging, or longitudinal monitoring remains to be established through prospective studies. Future large-scale, multicenter prospective studies are warranted to validate these findings and to explore longitudinal changes in the DTI-ALPS index and their relationship with cognitive outcomes.

## 5 Conclusion

In summary, this meta-analysis demonstrates that the DTI-ALPS index is significantly reduced in patients with CSVD, and that this reduction is associated with cognitive impairment. Similar reductions were observed across the examined CSVD subtypes. Notably, the DTI-ALPS index is already significantly reduced at the CSVD-MCI stage, with no further decline observed in CSVD-VaD in the limited available studies. Therefore, the current evidence supports an association between reduced DTI-ALPS indices and cognitive impairment in CSVD but does not establish its value for disease staging or progression. Despite the aforementioned limitations, the DTI-ALPS index s may represent non-invasive imaging metric associated with the presence of cognitive impairment. Future large-scale, multicenter prospective studies are warranted to further validate these findings and clarify the temporal relationship between alterations in the DTI-ALPS index and cognitive decline.
