# Supplementary material for: DTI-ALPS index and cognitive impairment in cerebral small vessel disease: a systematic review and meta-analysis
Source: Front Aging Neurosci. 2026 Aug 26;18:1853704. doi: 10.3389/fnagi.2026.1853704 (PMC13553545; doi:10.3389/fnagi.2026.1853704)
Supplement: Supplementary file 2 [file Table_2.docx]

**Pubmed search strategy**

#1 "Cerebral Small Vessel Diseases"[Mesh] OR "CSVD"[Title/Abstract] OR "Cerebral Microangiopath*"[Title/Abstract] OR "Small Vessel Disease*"[Title/Abstract] OR "SVD"[Title/Abstract] OR "White Matter Hyperintensit*"[Title/Abstract] OR "White Matter Lesion*"[Title/Abstract] OR "WML"[Title/Abstract] OR "WMH"[Title/Abstract] OR "Leukoaraiosis"[Mesh] OR "Leukoaraiosis"[Title/Abstract] OR "Lacunar infarct*"[Title/Abstract] OR "lacune*"[Title/Abstract] OR "Lacunar Stroke"[Title/Abstract] OR "Cerebral Microbleed*"[Title/Abstract] OR "Vascular Cognitive Impairment"[Title/Abstract] OR "VCI"[Title/Abstract] OR "Subcortical Vascular Dementia"[Title/Abstract]

#2 "Glymphatic System"[Mesh] OR "Glymphatic"[Title/Abstract] OR "Perivascular Space*"[Title/Abstract] OR "perivascular clearance"[Title/Abstract] OR "perivascular drainage"[Title/Abstract] OR "intramural peri-arterial drainage"[Title/Abstract] OR "IPAD"[Title/Abstract] OR "PVS"[Title/Abstract] OR "meningeal lymphatic"[Title/Abstract] OR "Virchow-Robin Space*"[Title/Abstract] OR "DTI-ALPS"[Title/Abstract] OR "ALPS index"[Title/Abstract] OR "Free Water"[Title/Abstract] OR "FW"[Title/Abstract] OR "Aquaporin 4"[Mesh] OR "AQP4"[Title/Abstract] OR "choroid plexus"[Title/Abstract] OR "ChP"[Title/Abstract] OR "enlarged perivascular space"[Title/Abstract] OR "EPVS"[Title/Abstract]

#3 #1 and #2

**Web of science search strategy**

#1 ((((((((((((((((TS=(Small Vessel Disease*)) OR TS=(CSVD)) OR TS=(Cerebral Microangiopath*)) OR TS=(SVD)) OR TS=(White Matter Hyperintensit*)) OR TS=(white matter lesion*)) OR TS=(WML)) OR TS=(WMH)) OR TS=(Leukoaraiosis)) OR TS=(Lacunar infarct*)) OR TS=(lacune*)) OR TS=(Lacunar Stroke)) OR TS=(CMB)) OR TS=(Vascular Cognitive Impairment)) OR TS=(VCI)) OR TS=(Subcortical Vascular Dementia)

#2 ((((((((((((((((((TS=(Glymphatic)) OR TS=(Perivascular Space*)) OR TS=(perivascular clearance)) OR TS=(perivascular drainage)) OR TS=(intramural peri-arterial drainage)) OR TS=(IPAD)) OR TS=(PVS)) OR TS=(meningeal lymphatic)) OR TS=(Virchow-Robin Space*)) OR TS=(DTI-ALPS)) OR TS=(ALPS index)) OR TS=(Free Water)) OR TS=(FW)) OR TS=(Aquaporin 4)) OR TS=(AQP4)) OR TS=(choroid plexus)) OR TS=(ChP)) OR TS=(enlarged perivascular space)) OR TS=(EPVS)

#3 #1 AND #2

**Embase search strategy**

#1 'cerebrovascular disease'/exp #2 csvd:ti,ab #3 'cerebral microangiopath*':ti,ab #4 'small vessel disease':ti,ab #5 'svd':ti,ab #6' white matter hyperintensit*':ti,ab #7' white matter lesion*':ti,ab #8' wml':ti,ab #9' wmh':ti,ab #10 'leukoaraiosis':ti,ab #11 'leukoaraiosis'/exp #12 'lacunar infarct*':ti,ab #13 'lacune*':ti,ab #14 'lacunar stroke':ti,ab #15' cerebral microbleed*':ti,ab #16 'vascular cognitive impairment':ti,ab# 17 'vci':ti,ab #18 'subcortical vascular dementia':ti,ab

#19: #1 OR #2 OR #3 OR #4 OR #5 OR #6 OR #7 OR #8 OR #9 OR #10 OR #11 OR #12 OR #13 OR #14 OR #15 OR #16 OR #17 OR #18

#20 'glymphatic system'/exp #21 'glymphatic':ti,ab #22 'perivascular space*':ti,ab #23 'perivascular clearance':ti,ab #24 'perivascular drainage':ti,ab #25 'intramural peri-arterial drainage':ti,ab #26 'ipad':ti,ab #27 'meningeal lymphatic':ti,ab #28 'virchow-robin space*':ti,ab #29 'dti-alps':ti,ab #30 'alps index':ti,ab #31 'free water':ti,ab #32 'fw':ti,ab #33 'aquaporin 4':ti,ab #34 'aqp4':ti,ab #35 'choroid plexus':ti,ab #36 'chp':ti,ab #37 'enlarged perivascular space':ti,ab #38 'epvs':ti,ab

#39 #20 OR #21 OR #22 OR #23 OR #24 OR #25 OR #26 OR #27 OR #28 OR #29 OR #30 OR #31 OR #32 OR #33 OR #34 OR #35 OR #36 OR #37 OR #38

#40 'cognition'/exp #41 cognition*:ti,ab #42 'cognitive function*':ti,ab #43 'insight':ti,ab #44 'cognitive':ti,ab #45 'memory':ti,ab #46 'executive function':ti,ab #47 'montreal cognitive assessment':ti,ab #48 'moca':ti,ab #49 'mini-mental state examination':ti,ab #50 'mmse':ti,ab

#51 #40 OR #41 OR #42 OR #43 OR #44 OR #45 OR #46 OR #47 OR #48 OR #49 OR #50

#52 #19 AND #39 AND #51

**Cochrane search strategy**

#1	MeSH descriptor: [Cerebral Small Vessel Diseases] explode all trees

#2	(CSVD):ti,ab,kw OR (Cerebral Microangiopath*):ti,ab,kw OR (Small Vessel Disease*):ti,ab,kw OR (SVD):ti,ab,kw OR (White Matter Hyperintensit*):ti,ab,kw

#3	(white matter lesion*):ti,ab,kw OR (WML):ti,ab,kw OR (WMH):ti,ab,kw

#4	MeSH descriptor: [Leukoaraiosis] explode all trees

#5	(Leukoaraiosis):ti,ab,kw OR (Lacunar infarct*):ti,ab,kw OR (lacune*):ti,ab,kw OR (Lacunar Stroke):ti,ab,kw OR (Cerebral Microbleed*):ti,ab,kw

#6	(Vascular Cognitive Impairment):ti,ab,kw OR (VCI):ti,ab,kw OR (Subcortical Vascular Dementia):ti,ab,kw

#7	#1 OR #2 OR #3 OR #4 OR #5 OR #6

#8	MeSH descriptor: [Glymphatic System] explode all trees

#9	(Glymphatic):ti,ab,kw OR (Perivascular Space*):ti,ab,kw OR (perivascular clearance):ti,ab,kw OR (perivascular drainage):ti,ab,kw OR (intramural peri-arterial drainage):ti,ab,kw

#10 (IPAD):ti,ab,kw OR (PVS):ti,ab,kw OR (meningeal lymphatic):ti,ab,kw OR (Virchow-Robin Space*):ti,ab,kw OR (DTI-ALPS):ti,ab,kw

#11 (ALPS index):ti,ab,kw OR (Free Water):ti,ab,kw OR (FW):ti,ab,kw OR (Aquaporin 4):ti,ab,kw OR (AQP4):ti,ab,kw

#12 (choroid plexus):ti,ab,kw OR (ChP):ti,ab,kw OR (enlarged perivascular space):ti,ab,kw OR (EPVS):ti,ab,kw

#13 #8 OR #9 OR #10 OR #11 OR #12

#14 #7 AND #13

**China Knowledge Network (CNKI) search strategy**

#1主题：脑小血管病 + 脑小血管疾病 + 脑小血管病变 + 脑微血管病 + 脑微血管疾病 + 脑白质高信号 + 白质高信号 + 脑白质病变 + 白质病变 + 脑白质疏松 + 白质疏松 + 腔隙性梗死 + 腔隙性脑梗死 + 腔隙性卒中 + 腔梗 + 脑微出血 + 微出血 + 血管性认知损害 + 血管性认知障碍 + 血管性痴呆 + 皮质下血管性痴呆

#2主题：类淋巴系统 + 类淋巴 + 胶质淋巴系统 + 胶质淋巴 + 血管周围间隙 + 血管周围引流 + 血管周围清除 + 血管周隙 + Virchow-Robin间隙 + 弗尔绍-罗宾间隙 + 沿血管周围间隙扩散张量分析 + DTI-ALPS + ALPS指数 + 自由水 + 自由水分数 + 水通道蛋白4 + AQP4 + 水通道蛋白-4 + 脉络丛 + 脑膜淋巴 + 脑膜淋巴管 + 扩大的血管周围间隙 + EPVS

#3 #1 AND #2

**WangFang Data search strategy**

#1题名或主题词： 脑小血管病 OR 脑微血管病 OR 白质高信号 OR 白质疏松 OR 腔隙性梗死 OR 腔梗 OR 脑微出血 OR 血管周围间隙 OR 血管性认知障碍 OR 血管性痴呆

#2题名或主题词：类淋巴 OR 血管周围间隙 OR Virchow-Robin间隙 OR DTI-ALPS OR ALPS指数 OR 自由水 OR AQP4 OR 脉络丛 OR EPVS

#3 #1 AND #2

**SinoMed search strategy**

#1题名或主题词： ("大脑小血管疾病"[不加权:扩展] OR "脑白质高信号"[常用字段:智能] OR "白质高信号"[常用字段:智能] OR "腔隙性梗死"[常用字段:智能] OR "脑微出血"[常用字段:智能] OR "血管周围间隙"[常用字段:智能] OR "血管性认知障碍"[不加权:扩展] OR "血管性痴呆"[不加权:扩展])

#2题名或主题词： ("类淋巴系统"[常用字段:智能] OR "血管周围间隙"[常用字段:智能] OR "水通道蛋白4"[常用字段:智能] OR "脉络丛"[不加权:扩展] OR "DTI-ALPS"[常用字段:智能] OR "自由水"[常用字段:智能])

#3 #1 AND #2

**VIP Chinese Journal Database (VIP, Qikan) search strategy**

#1：[(题名或关键词=脑小血管病 + 脑小血管疾病 + 脑小血管病变 + 脑微血管病 + 脑微血管疾病 + 脑白质高信号 + 白质高信号 + 脑白质病变 + 白质病变 + 脑白质疏松 + 白质疏松 + 腔隙性梗死 + 腔隙性脑梗死 + 腔隙性卒中 + 腔梗 + 脑微出血 + 微出血 + 血管性认知损害 + 血管性认知障碍 + 血管性痴呆 + 皮质下血管性痴呆)](https://ras.cdutcm.edu.cn:7080/s/com/cqvip/qikan/G.http/Qikan/search/index?LngMySearHistoryIdGuid=04101bb2-983e-440c-8e4f-0ee5cb38ac39&from=Qikan_Article_History)

#2：(题名或关键词=类淋巴系统 + 类淋巴 + 胶质淋巴系统 + 胶质淋巴 + 血管周围间隙 + 血管周围引流 + 血管周围清除 + 血管周隙 + Virchow-Robin间隙 + 弗尔绍-罗宾间隙 + 沿血管周围间隙扩散张量分析 + DTI-ALPS + ALPS指数 + 自由水 + 自由水分数 + 水通道蛋白4 + AQP4 + 水通道蛋白-4 + 脉络丛 + 脑膜淋巴 + 脑膜淋巴管 + 扩大的血管周围间隙 + EPVS)

#3 #1 AND #2
