# Supplementary material for: DTI-ALPS index and cognitive impairment in cerebral small vessel disease: a systematic review and meta-analysis
Source: Front Aging Neurosci. 2026 Aug 26;18:1853704. doi: 10.3389/fnagi.2026.1853704 (PMC13553545; doi:10.3389/fnagi.2026.1853704)
Supplement: Supplementary file 3 [file Table_3.docx]

**Supplementary Table S3.**

Assessment of potential cohort overlap among included studies.

| First Author, Year | Institution | City/Province | Recruitment Period | Sample Source | CSVD Sample Size | HC Sample Size | Potential Cohort Overlap | Notes |
| --- | --- | --- | --- | --- | --- | --- | --- | --- |
| Jiajie Xu, 2022 | Huashan Hospital, Fudan University | Shanghai | January 2014 – September 2019 | Hospital-based | 63 | 70 | Evaluated | CAA-specific cohort with a clearly reported recruitment period. No evidence of participant overlap was identified. |
| Jie Tang, 2022 | Huashan Hospital, Fudan University | Shanghai | NR | Hospital-based | 133 | — | Evaluated | Same institution as Jiajie Xu (2022) and Yilei Chen (2025). Comparison of study population, participant characteristics, inclusion criteria, and study objectives revealed no evidence of participant overlap. |
| Zhihong Ke, 2022 | Drum Tower Hospital, Nanjing University | Nanjing, Jiangsu | NR | Hospital-based | 137 | 52 | No evidence identified | Different institution and study population. No evidence of overlapping participants. |
| Qin Zhong, 2024 | Wuhan Central Hospital, Huazhong University of Science and Technology | Wuhan, Hubei | June 2022 – March 2023 | Hospital-based + Community | 70 | 37 | No evidence identified | Different institution with clearly reported recruitment period. |
| Yanan Xu, 2024 | ZhongDa Hospital, Southeast University | Nanjing, Jiangsu | NR | Hospital-based | 206 | 43 | No evidence identified | Different institution from other Nanjing cohorts. No evidence of overlapping participants. |
| Lulu Ai, 2025 | The First Affiliated Hospital of Anhui Medical University | Hefei, Anhui | NR | Hospital-based | 120 | 40 | No evidence identified | Different institution. No evidence of overlapping participants. |
| Yage Qiu, 2025 | Ren Ji Hospital, Shanghai Jiao Tong University | Shanghai | NR | Hospital-based | 102 | 29 | No evidence identified | Different institution from Huashan Hospital. |
| Min Xu, 2025 | The Affiliated Wuxi People's Hospital of Nanjing Medical University | Wuxi, Jiangsu | December 2021 – January 2024 | Hospital-based + Community | 160 | 190 | No evidence identified | Different institution with reported recruitment period. |
| Mingming Huang, 2025 | The Affiliated Hospital of Guizhou Medical University | Guiyang, Guizhou | January 2020 – January 2023 | Hospital-based | 72 | 40 | No evidence identified | Different institution with reported recruitment period. |
| Yijun Lin, 2025 | Beijing Tiantan Hospital, Capital Medical University | Beijing | January 2022 – June 2024 | Hospital-based | 64 | 47 | No evidence identified | Different institution with reported recruitment period. |
| Bang Zeng, 2025 | The First Affiliated Hospital of Chongqing Medical University | Chongqing | February 2023 – August 2024 | Hospital-based | 84 | 23 | No evidence identified | Different institution with reported recruitment period. |
| Wenli Lu, 2025 | BenQ Medical Center, Nanjing Medical University | Nanjing, Jiangsu | NR | Hospital-based + Community | 102 | 29 | No evidence identified | Different institution from Drum Tower Hospital and ZhongDa Hospital. No evidence of overlapping participants. |
| Jinghan Zhao, 2025 | The First Affiliated Hospital of Dalian Medical University | Dalian, Liaoning | February 2023 – May 2024 | Hospital-based | 81 | 20 | No evidence identified | Different institution with reported recruitment period. |
| Yilei Chen, 2025 | Huashan Hospital, Fudan University | Shanghai | NR | Hospital-based | 135 | — | Evaluated | Same institution as Jiajie Xu (2022) and Jie Tang (2022). Comparison of study population, participant characteristics, inclusion criteria, and available study information revealed no evidence of participant overlap. |

NR, not reported (the information was not provided in the original publication); —, not applicable (no healthy control group was included in the study). Potential cohort overlap was evaluated by comparing the recruitment periods, institutions, author lists, study populations, inclusion criteria, participant characteristics, and sample sizes of all included studies. For studies conducted at the same institution (Huashan Hospital: Jiajie Xu 2022, Jie Tang 2022, and Yilei Chen 2025), further detailed comparisons of sample characteristics and inclusion criteria were performed, and no evidence of participant overlap was identified. Where recruitment periods were not reported, the assessment was based on all other available study characteristics. No evidence of duplicate participant inclusion was identified among the studies included in the final meta-analysis.
