# Supplementary material for: DTI-ALPS index and cognitive impairment in cerebral small vessel disease: a systematic review and meta-analysis
Source: Front Aging Neurosci. 2026 Aug 26;18:1853704. doi: 10.3389/fnagi.2026.1853704 (PMC13553545; doi:10.3389/fnagi.2026.1853704)
Supplement: Supplementary file 4 [file Table_4.docx]

**Supplementary Table S4.**

Diagnostic criteria for cerebral small vessel disease (CSVD) across

| First Author, Year | CSVD Subtype | Diagnostic Criteria |
| --- | --- | --- |
| Jiajie Xu, 2022 | CAA | Modified Boston criteria for probable CAA (CAA-ICH or CAA-related cognitive decline). |
| Jie Tang, 2022 | Sporadic CSVD | STRIVE criteria; Fazekas grade 2–3 WMH plus ≥1 additional CSVD marker (lacune, cerebral microbleed, or enlarged perivascular space). |
| Yage Qiu, 2025 | Sporadic CSVD | STRIVE-2 criteria; MRI-confirmed CSVD neuroimaging markers. |
| Yanan Xu, 2024 | WMH | White matter hyperintensities defined by Fazekas score ≥1 on FLAIR MRI. |
| Bang Zeng, 2025 | WMH | STRIVE criteria; WMH as the primary CSVD manifestation, with total WMH volume ≤10 mL and WMH count ≤20. |
| Jinghan Zhao, 2025 | Sporadic CSVD | STRIVE-2 criteria; lacunar syndrome with subcortical infarction and/or WMH (Fazekas score ≥2). |
| Lulu Ai, 2025 | Sporadic CSVD | STRIVE criteria (Wardlaw et al., 2013); total CSVD burden score ≥1. |
| Min Xu, 2025 | Sporadic CSVD | STRIVE-2 criteria; summary CSVD burden score >1 with MRI findings consistent with CSVD. |
| Mingming Huang, 2025 | Sporadic CSVD | 2016 AHA/ASA criteria; MRI evidence of moderate-to-severe WMH (Fazekas 2–3), lacunes (<15 mm), with or without enlarged perivascular spaces, cerebral microbleeds, and brain atrophy. |
| Qin Zhong, 2024 | Sporadic CSVD | STRIVE criteria; Fazekas score ≥2, or Fazekas score = 1 with ≥1 vascular risk factor (hypertension, diabetes, hyperlipidemia, hyperhomocysteinemia, smoking, or alcohol consumption). |
| Wenli Lu, 2025 | Sporadic CSVD | STRIVE-2 criteria; total CSVD imaging burden score ≥1. |
| Yijun Lin, 2025 | CAA | Boston criteria version 2.0 for probable CAA-ICH. |
| Yilei Chen, 2025 | Sporadic CSVD | STRIVE-2 criteria; MRI-confirmed CSVD neuroimaging markers. |
| Zhihong Ke, 2022 | Sporadic CSVD | STRIVE criteria; Fazekas grade 2–3 WMH and/or lacunes, with or without enlarged perivascular spaces, cerebral microbleeds, and brain atrophy. |

AHA/ASA, American Heart Association/American Stroke Association; CAA, cerebral amyloid angiopathy; CAA-ICH, cerebral amyloid angiopathy-related intracerebral hemorrhage; CSVD, cerebral small vessel disease; FLAIR, fluid-attenuated inversion recovery; MRI, magnetic resonance imaging; STRIVE, Standards for Reporting Vascular Changes on Neuroimaging; WMH, white matter hyperintensity.
