# Supplementary material for: DTI-ALPS index and cognitive impairment in cerebral small vessel disease: a systematic review and meta-analysis
Source: Front Aging Neurosci. 2026 Aug 26;18:1853704. doi: 10.3389/fnagi.2026.1853704 (PMC13553545; doi:10.3389/fnagi.2026.1853704)
Supplement: Supplementary file 5 [file Table_5.docx]

| Comparison | Primary Model | Primary Pooled Estimate | Fixed-Effects Sensitivity Analysis | I² | Consistency |
| --- | --- | --- | --- | --- | --- |
| CSVD-MCI vs CSVD-NC | Random-effects | WMD = −0.07 (95% CI −0.10 to −0.05), P < 0.00001 | WMD = −0.07 (95% CI −0.10 to −0.05), P < 0.00001 | 0% | No material change |
| CSVD-VaD vs CSVD-NC | Random-effects | WMD = −0.13 (95% CI −0.20 to −0.07), P < 0.0001 | WMD = −0.13 (95% CI −0.18 to −0.08), P < 0.00001 | 34% | No material change |
| CSVD-VaD vs CSVD-MCI | Random-effects | WMD = −0.03 (95% CI −0.08 to 0.02), P = 0.19 | WMD = −0.03 (95% CI −0.08 to 0.02), P = 0.19 | 0% | No material change |

**Supplementary Table S5.**

Sensitivity analyses using fixed-effects models for comparisons with low heterogeneity

Sensitivity analyses using fixed-effects models were conducted for comparisons with low heterogeneity (I² < 50%). The pooled effect estimates showed no meaningful differences compared with the primary random-effects analyses, supporting the robustness of the findings. CSVD, cerebral small vessel disease; MCI, mild cognitive impairment; NC, normal cognition; VaD, vascular dementia; WMD, weighted mean difference; CI, confidence interval.
