# Supplementary material for: DTI-ALPS index and cognitive impairment in cerebral small vessel disease: a systematic review and meta-analysis
Source: Front Aging Neurosci. 2026 Aug 26;18:1853704. doi: 10.3389/fnagi.2026.1853704 (PMC13553545; doi:10.3389/fnagi.2026.1853704)
Supplement: Supplementary file 6 [file Table_6.docx]

**Supplementary Table S6.**

Imaging acquisition and DTI-ALPS processing characteristics of the included studies.

| First Author, Year | Preprocessing | Tensor Fitting | ROI Placement Method | ROI Size | ROI Laterality | Inter-rater Reliability | Ventricular / Atrophy Correction | ALPS Calculation |
| --- | --- | --- | --- | --- | --- | --- | --- | --- |
| Jiajie Xu, 2022 | DTI-Studio software | DTI-Studio software | Manual; projection and association fibers | 5-mm sphere | Left hemisphere (non-hemorrhagic side) | Two independent raters (average values reported); ICC not reported | NR | Left hemisphere only |
| Jie Tang, 2022 | FSL (eddy-current and motion correction) | FSL (FDT) | Manual; projection, association, and subcortical fibers | 5-mm sphere | Left hemisphere | NR | NR | Left hemisphere only |
| Zhihong Ke, 2022 | FSL (affine registration and eddy-current correction) | FSL (dtifit) * | Manual; projection and association fibers | 12-voxel sphere | Left hemisphere | NR | NR | Left hemisphere only |
| Qin Zhong, 2024 | MRtrix3 (denoising, Gibbs ringing removal, motion/eddy-current correction, bias-field correction) | FSL | Manual; projection and association fibers | 5-mm sphere | Bilateral | NR | NR | Bilateral (averaged) |
| Yanan Xu, 2024 | FSL (eddy-current and motion correction) | FSL | Automated (bash script) with manual verification; JHU-ICBM-DTI-81 atlas | 5-mm sphere | Bilateral | Inter-method ICC = 0.936 | NR | Bilateral (averaged) |
| Lulu Ai, 2025 | FSL (motion and eddy-current correction) | FSL | Manual; projection and association fibers | 5-mm sphere | Left hemisphere | Inter-rater ICC = 0.94; Intra-rater ICC = 0.96 | NR | Left hemisphere only |
| Yage Qiu, 2025 | FSL (EPI distortion correction, eddy-current and motion correction) | FSL | Semi-automated; JHU-ICBM-DTI-81 atlas with manual adjustment to avoid WMH | 5-mm sphere | Bilateral | NR | NR | Bilateral (averaged) |
| Min Xu, 2025 | FSL | FSL | Manual; projection and association fibers | 3-mm sphere | Bilateral | NR | NR | Bilateral (averaged) |
| Mingming Huang, 2025 | FSL (denoising, Gibbs ringing removal, motion/distortion correction, bias-field correction) | FSL | Manual; projection and association fibers | 5-mm sphere | Bilateral | NR | NR | Bilateral (averaged) |
| Yijun Lin, 2025 | FSL (eddy-current and motion correction) | FSL | Automated; MNI-space template; projection and association fibers | 6-mm sphere | Bilateral | NR | NR | Bilateral mean ALPS (I-ALPS and C-ALPS additionally calculated) |
| Bang Zeng, 2025 | FSL (eddy-current and motion correction) | FSL | Automated (Auto DTI-ALPS pipeline) with manual repositioning | NR | Bilateral | NR | NR | Bilateral (averaged) |
| Wenli Lu, 2025 | MRtrix3 (denoising, Gibbs ringing removal, motion/eddy-current correction, bias-field correction) | FSL (dtifit) | Manual; projection and association fibers | 3-mm sphere | Bilateral | NR | NR | Bilateral (averaged) |
| Jinghan Zhao, 2025 | FSL (head motion, eddy-current, and gradient direction correction) | FSL (dtifit) | Manual; projection and association fibers | 2.5-mm-radius sphere | Left hemisphere | Inter-rater ICC = 0.859 | NR | Left hemisphere only |
| Yilei Chen, 2025 | DSI Studio (phase distortion mitigation, eddy-current and motion correction) | DSI Studio | Manual; projection and association fibers | 5-mm square ROI | Bilateral | Inter-rater ICC = 0.901 (left); 0.886 (right) | NR | Bilateral (averaged) |

DTI-ALPS processing and quality assessment characteristics of studies included in the meta-analysis. MRI acquisition parameters (field strength, b-value, diffusion directions, and coil type) are presented in Table 1. DTI, diffusion tensor imaging; ROI, region of interest; ICC, intraclass correlation coefficient; NR, not reported; FSL, FMRIB Software Library; EPI, echo-planar imaging; WMH, white matter hyperintensity; MNI, Montreal Neurological Institute; I-ALPS, ipsilateral ALPS index; C-ALPS, contralateral ALPS index.
